# Machine learning analysis of posturography in panic disorder: a pilot study for objective physiological biomarker identification

**DOI:** 10.3389/fpsyt.2025.1663556

**Published:** 2025-10-16

**Authors:** Luiz Antonio Vesco Gaiotto, Felipe O. Aguiar, Thales Marcon, Julia Souza Gallo, Lucas Murrins Marques, Ricardo R. Uchida

**Affiliations:** ^1^ Mental Health Department, Santa Casa de São Paulo School of Medical Sciences, São Paulo, Brazil; ^2^ Artificial Intelligence Research Division, Infinity Doctor’s Inc., DE, Miami, FL, United States; ^3^ Santa Casa de São Paulo School of Medical Sciences, São Paulo, Brazil

**Keywords:** panic disorder, posturography, machine learning, physiological biomarkers, postural control

## Abstract

Panic disorder (PD) is linked to subtle abnormalities in postural control, which are inadequately captured by traditional statistics. Machine learning (ML) techniques applied to stabilometric data may enhance the detection of PD-related postural patterns.

**Objective:**

Evaluate static postural control in PD patients and determine if ML analysis of multivariate stabilometric data can improve differentiation from healthy controls.

**Methods:**

In this cross-sectional case-control study, 12 adults diagnosed with DSM-5 PD and 21 matched healthy volunteers (total n = 33; 341 force platform trials) underwent stabilometry under five sensory conditions. Classical statistics used repeated-measures ANOVA on baseline trials only (to preserve independence). ML models (Decision Tree, k-Nearest Neighbors, Linear Discriminant Analysis, Logistic Regression, and Random Forest) were trained under stratified, subject-grouped, fourfold cross-validation (StratifiedGroupKFold), ensuring that all trials from each participant were confined to a single fold to prevent leakage. For the explainability of the model, Local Interpretable Model-Agnostic Explanations (LIME) was accessed.

**Results:**

ANOVA revealed a significant group and condition interaction for mediolateral center of pressure (CoP) displacement (p < 0.01), with PD patients exhibiting consistently reduced mediolateral sway. No significant between-group differences emerged for anteroposterior sway. Using an optimized decision threshold (Youden), Logistic Regression achieved an accuracy of 93.8% and area under the receiver operating characteristic curve (AUC) = 96%; Linear Discriminant Analysis presented the highest specificity (91.7%).

**Conclusions:**

This is the first study applying ML to posturography for identifying physiological markers of panic disorder. Using ML for stabilometric data improves classification accuracy, highlighting static posturography as superior to clinical screening tools like the Patient Health Questionnaire for Panic Disorder (PHQ-PD) and Panic Disorder Severity Scale (PDSS). Larger, externally validated cohorts and portable measurement solutions are needed to translate these findings into routine clinical assessment.

## Introduction

Panic disorder (PD) is a prevalent anxiety disorder that imposes a substantial individual and societal burden, with its manifestations extending beyond the acute panic attack with neurobiological and behavioral changes that reflect underlying dysfunctions in stress response systems. A range of physiological changes have been documented in individuals with PD who not only experience the hallmark symptoms of acute panic attacks and persistent anticipatory anxiety but also exhibit chronic alterations in bodily regulation that extend far beyond the acute episodes. These physiological changes include, but are not limited to, chronic hyperventilation, irregular respiratory patterns, cardiorespiratory instabilities, and a marked increase in the frequency and severity of dizziness, vestibular complaints, and dysfunctions (1–6).

Symptoms such as non-specific dizziness, a sensation of floating, or instability are frequently reported by patients with PD and can be exacerbated in environments with conflicting sensory cues, such as crowds, stairs, or high places. It has been shown that these complaints are not always correlated with identifiable peripheral vestibular dysfunction (7, 8). A recent meta-analysis of panic disorder in the population with vestibular dysfunctions found an increased prevalence of PD versus the control group (9).

Individuals with PD, in addition to affective and autonomic symptoms, show subtle yet relevant alterations in sensory processing and integration related to body balance control (5, 7, 10). Such alterations suggest a dysfunction in integrating information from the vestibular, visual, and somatosensory systems, which are responsible for maintaining an upright posture at rest and during movement (2, 10–12).

Functional neuroimaging studies demonstrate that brain circuits involved in emotional regulation and fear response, such as the amygdala, hippocampus, insula, and medial prefrontal cortex, also influence postural motor control, as they share connections with vestibular nuclei and brainstem structures (5, 10, 12) Altered activation and/or connectivity between regions linked to sensory processing in patients with PD may contribute to increased vigilance, and these neuroanatomical substrates also interact dynamically during the maintenance of posture and in the modulation of balance responses to both external and internal stimuli (10, 13).

The assessment of static postural control is a possible physiological marker for PD. Stabilometric analysis using force platforms that record the displacement of the center of pressure (CoP) allows for the measurement of postural sway and the evaluation of postural control strategies under different sensory conditions (14). This method has proven sensitive in detecting altered balance patterns in individuals with mental disorders, suggesting its potential as a diagnostic and monitoring tool (15–21).

However, data derived from force platforms are multivariate and dynamic, requiring robust analytical approaches to extract clinically relevant information. In this context, the application of machine learning (ML) techniques emerges as an innovative and promising approach. Machine learning methods, such as Linear Discriminant Analysis, Logistic Regression, and Random Forest, show advantages in classification and prediction capabilities when dealing with complex and non-linear physiological patterns (22, 23).

This study aimed to evaluate static postural control in individuals with panic disorder and investigate whether applying machine learning algorithms to multivariate stabilometric data can enhance the ability to discriminate between patients and healthy controls. We believe that this approach can significantly contribute to identifying physiological markers of PD, with relevant clinical and neurobiological implications.

## Methods

### Study design

This was a cross-sectional, case–control study conducted between March 2022 and January 2023 at the Mental Health Department of Santa Casa de São Paulo School of Medical Sciences. The objective was to evaluate the postural control of individuals diagnosed with PD, comparing them to healthy controls, through stabilometric analysis. The protocol was approved by the institution’s Research Ethics Committee (CAAE:09729019.1.0000.5479).

### Participants

The sample consisted of 33 adults: 12 individuals with a diagnosis of PD and 21 healthy controls matched for age and sex. Participants in the PD group were recruited through institutional social media campaigns and contacted by the research team via email. All procedures were conducted after informed consent was obtained.

The inclusion criteria for the PD group included the following: 1) a diagnosis of panic disorder confirmed by the Structured Clinical Interview for DSM-5 Disorders, Clinician Version (SCID-5-CV), and 2) a Panic Disorder Severity Scale (PDSS) score >8. The exclusion criteria included the following: 1) comorbid psychiatric diagnoses such as bipolar disorder, psychotic disorders, substance use disorder, or neurodevelopmental conditions, and 2) vestibular dysfunction or self-reported hearing impairment.

A total of 341 posturographic trials were collected using a force platform (described below). Patients in the PD group underwent three repeated stabilometric assessments—baseline, after 1 hour, and after 1 week—in order to expand the dataset for machine learning analysis. Only the baseline trial was used for conventional statistical analysis to ensure data independence.

### Clinical assessment

The diagnostic evaluation was conducted using the SCID-5-CV (24), administered by board-certified psychiatrists trained in its application. The SCID-5-CV is considered the gold standard for structured psychiatric diagnosis and ensures high inter-rater reliability.

To assess the severity of panic symptoms, the PDSS (25) was used. This scale has demonstrated good psychometric properties and is widely employed in both clinical and research settings to quantify the intensity and functional impact of panic-related symptoms. The version used was the officially translated and culturally adapted Brazilian Portuguese version.

### Posturographic assessment

All participants underwent static posturographic assessment using a digital triaxial force platform P–6000 (BTS Bioengineering S.p.A., Garbagnate Milanese, Italy) (26) with a sampling frequency of 10 Hz. Each participant completed five trials under distinct sensory conditions designed to progressively challenge postural control mechanisms. In the first condition (P1), participants remained with their eyes open on a firm surface, providing full access to visual and plantar somatosensory input. In the second condition (P2), they stood on a foam surface with eyes open, reducing the reliability of somatosensory input from the feet while maintaining visual cues. The third condition (P3) involved closing the eyes on a firm surface, thereby removing visual input and requiring greater reliance on somatosensory and vestibular systems. In the fourth condition (P4), participants stood on the foam surface with eyes closed, simultaneously removing visual input and degrading plantar somatosensory feedback. Finally, in the fifth condition (P5), the most demanding, participants stood on the foam surface with eyes closed while engaging in a standardized verbal interaction, adding a cognitive distraction to the dual sensory challenge.

During the distraction condition (P5), a standardized conversational script was administered by a trained psychiatrist positioned directly in front of the participant to impose a dual-task demand on attention and postural control. This condition was designed to simulate cognitive load while proprioceptive and visual input were reduced. In all conditions, participants stood barefoot, with feet positioned hip–width apart (approximately 10 cm between heels) and arms relaxed at their sides. Each trial lasted 30 seconds, a duration commonly used in static posturographic evaluations to ensure reliable CoP measurements while minimizing fatigue.

### Analyzed variables

For the machine learning data analysis, we used all variables extracted from the force platform data using the SWAY software (27) (**Table 1**).

**Table 1:** Posturographic variables exported by SWAY (BTS Bioengineering).

Transversal CoP displacement (mm)	Equivalent radius (mm)	Peak number, peak amplitude (s)
Longitudinal CoP displacement (mm)	Speed (mm/s) [m, sd]	Peak time (s)
Radius (mm) [m, sd]	Inertial axes (mm) [asseX, asseY]	Peak distance (mm) [m, sd]
Max radius (mm)	Regression angle (°)	Px spectrum, maximum peak, and frequency (Hz)
Min radius (mm)	Geographic area (mmq)	Py spectrum, maximum peak, and frequency (Hz)
Transversal range (mm)	Fixed mean area (mmq)	D spectrum, maximum peak, and frequency (Hz)
Longitudinal range (mm)	Fixed sum area (mmq)	Px spectrum, mean values
Trace length (mm)	Variable mean area (mmq)	Py spectrum, mean values
LFS (1/cm)	Variable sum area (mmq)	D spectrum, mean values
Equivalent area (mmq)		

CoP, center of pressure; Px/Py/D spectrum, power spectral metrics of CoP in the mediolateral (x), anteroposterior (y), and resultant distance signals; LFS, software-reported length/space frequency score (unit 1/cm); m, mean; sd, standard deviation; Hz, hertz; mm, millimeters; mm^2^, square millimeters; mm/s, millimeters per second; °, degrees.

### Statistical analysis

Data were first tested for normality using the Shapiro–Wilk test. Descriptive statistics were calculated for all variables. Group differences (panic disorder vs. controls), condition effects (five sensory conditions), and group-by-condition interactions were analyzed using repeated-measures ANOVA (RM-ANOVA), with group as a between-subjects factor and condition as a within-subjects factor. The analysis focused on four key stabilometric variables: trace length, mean velocity, mediolateral displacement, and anteroposterior displacement. The significance threshold was set at p < 0.05. All statistical analyses were conducted using the JASP software (28). For these classical analyses, only the baseline trial for each participant was included; repeated assessments used to enrich the machine learning dataset were excluded to ensure the independence of observations.

### Machine learning analysis

All 28 features exported using the SWAY software (27) from each of the 341 posturographic trials were included without feature selection to preserve the full dimensionality of the posturographic signal. To make a model with better real-world usability, we considered a single posturographic instance per participant in the held-out test folds. Model development employed five classical algorithms with diverse decision strategies and interpretability: Decision Tree (DT), k–Nearest Neighbors (KNN), Linear Discriminant Analysis (LDA), Logistic Regression (LR), and Random Forest (RF) (scikit–learn and Python). Because features had varying numerical ranges, data were standardized for the KNN, LDA, and LR models using StandardScaler (zero mean and unit variance).

We used StratifiedGroupKFold (K = 4) with class stratification in every fold, grouping by participant during partitioning, so that all trials from a given individual are assigned to a single fold (i.e., never split across training and test), avoiding subject-level leakage and thereby reducing overfitting risk.

Splits were shuffled with a fixed random state. Primary validation metrics [F1–score and area under the receiver operating characteristic curve (AUC)] were computed on the held-out split of each fold and averaged as mean ± SD across folds. The full hyperparameter search spaces and the best-performing settings for each model are provided in **Supplementary Table S1**. , 

Given the presence of class imbalance, stratification preserved class proportions, and reliance on the F1–score and AUC as primary validation metrics appropriately addressed imbalance during cross-validation while preserving the empirical distribution. Hyperparameters were optimized within each training fold via grid search using the F1-score as the objective.

Two decision thresholds were reported: a baseline threshold of 0.50 and a Youden’s J-optimized cutoff (sensitivity + specificity − 1) estimated on the training split and applied to the test split of each fold.

#### Model interpretability

Variable importance was explored using Local Interpretable Model-Agnostic Explanations (LIME) to identify features contributing the most to model decisions. A total of 28 features extracted using the SWAY software from each of the 341 posturographic trials were included in the dataset without additional feature selection in order to preserve the full dimensionality of posturographic data.

## Results

**Figure 1:**
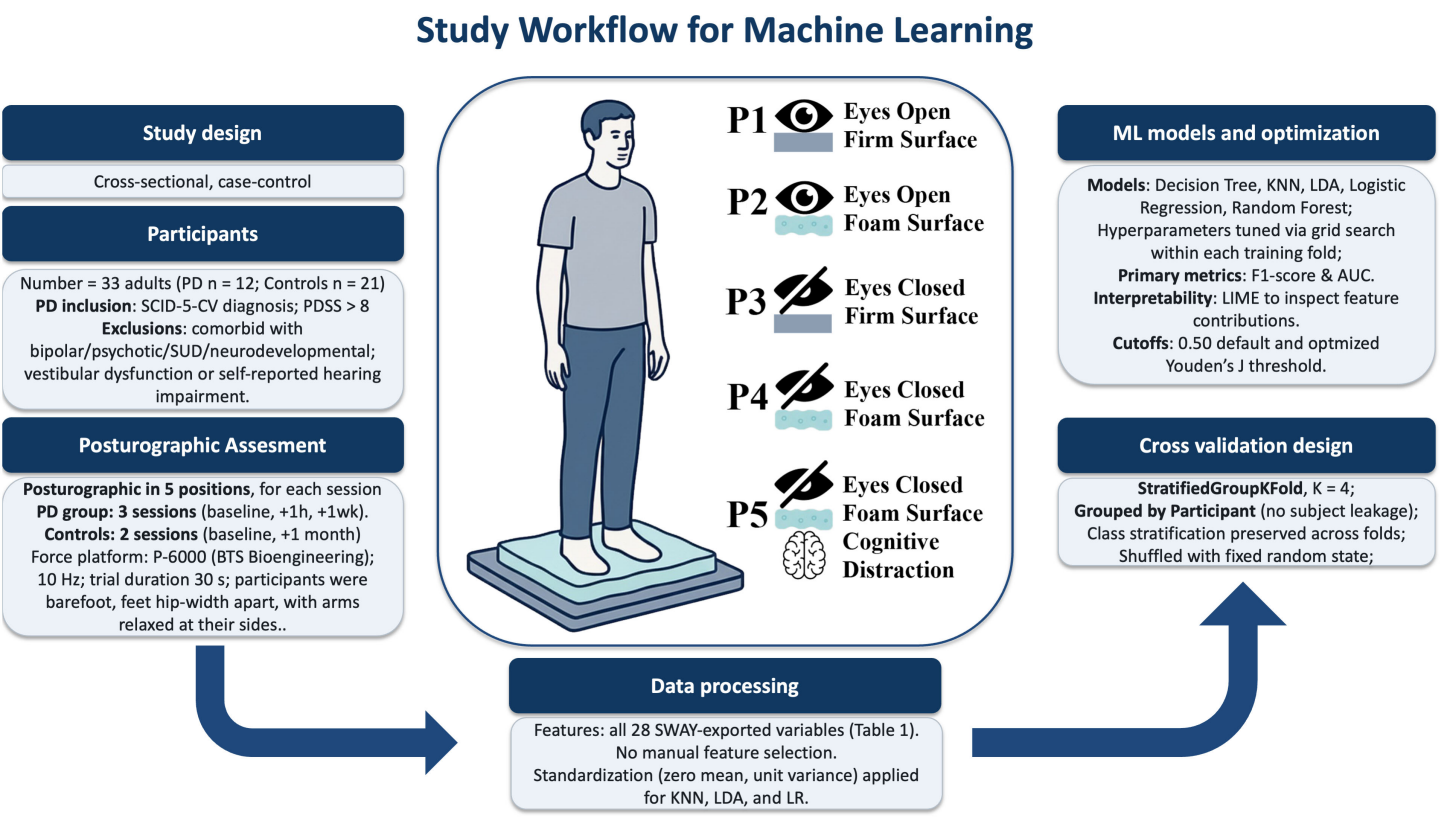
Posturographic assessment and study workflow for machine learning analysis of stabilometry in panic disorder. PD, panic disorder; SCID-5-CV, Structured Clinical Interview for DSM-5, Clinician Version; PDSS, Panic Disorder Severity Scale; SUD, substance use disorder; Hz, hertz; s, seconds; SWAY, BTS Bioengineering software; KNN, k-Nearest Neighbors; LDA, Linear Discriminant Analysis; LR, Logistic Regression; AUC, area under the ROC curve; LIME, Local Interpretable Model-Agnostic Explanations.

Descriptive statistics for age and education in both groups, as well as panic disorder severity (measured using the PDSS), the presence of psychiatric comorbidities, and current pharmacological treatments, are presented in **Table 2**. An RM-ANOVA was performed on four variables: mediolateral CoP displacement, anteroposterior CoP displacement, trace length, and mean velocity (see **Figure 1** for the descriptive plots from the RM-ANOVA).

**Table 2:** Population description.

	Panic disorder group	Control group
Number of individuals	12	21
Median age	43	20
(Minimum, maximum)	(min. 22, max. 62)	(min. 19, max. 24)
Median years of education	15	14
Medium score PDSS	14	–
Number of individuals	Severe: 6	–
Severity of PDSS score	Moderate: 3	
	Mild: 3	
Comorbid with agoraphobia	6	–
Psychiatric comorbidity	MDD: 4	–
	GAD: 2	
Drugs in use	SSRI: 1	–
	SSRI + GBP: 1	
	SSRI + BZD + buspirone: 1	
	SSRI + TCA: 1	

PDSS, Panic Disorder Severity Scale; MDD, major depressive disorder; GAD, generalized anxiety disorder; SSRI, selective serotonin reuptake inhibitor; GBP, gabapentinoid (pregabalin); BZD, benzodiazepine; TCA, tricyclic antidepressant.

### Mediolateral CoP displacement

**Figure 2:**
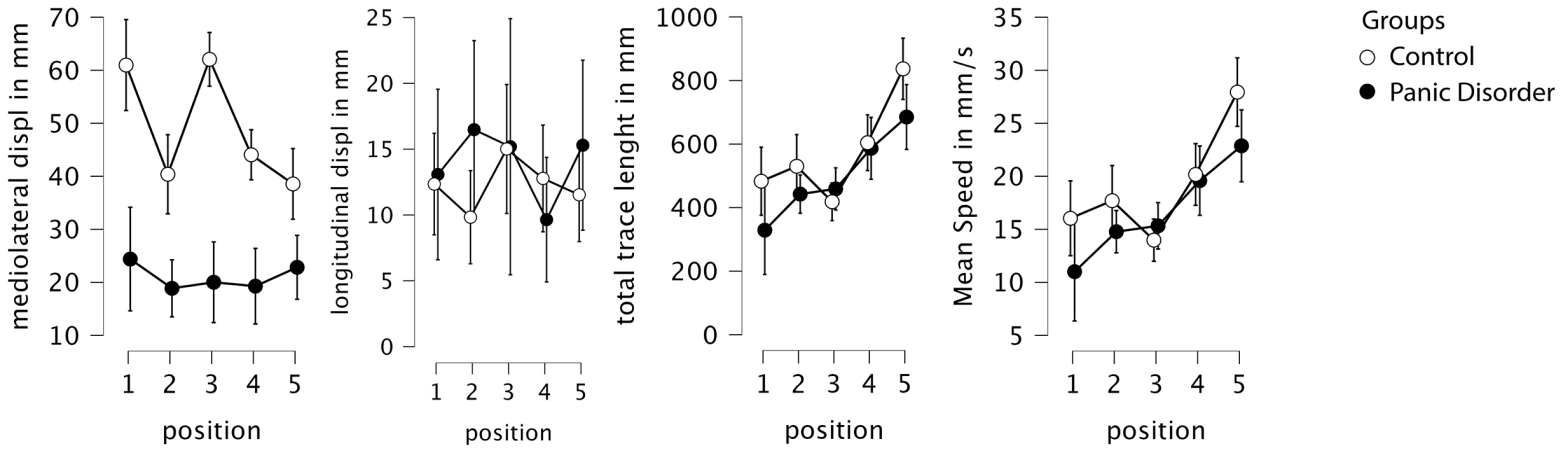
Descriptive plots from the RM-ANOVA, with 95% confidence interval. Displ, displacement; mm, millimeter; s, seconds; RM-ANOVA, repeated-measures ANOVA.

A repeated-measures ANOVA was conducted to analyze mean mediolateral (transversal) displacement across five postural conditions between groups. The analysis revealed a significant main effect of condition (F = 7.05; p < 0.001; η^2^ = 0.050), indicating that the type of sensory challenge influenced postural sway. There was also a significant main effect of group (F = 21.83; p < 0.001; η^2^ = 0.322), with the control group showing substantially higher overall sway values than the PD group. Furthermore, a significant group-by-condition interaction was observed (F = 5.43; p < 0.001; η^2^ = 0.039), suggesting that the pattern of sway across conditions differed between groups (**Figure 2**).

**Figure 3:**
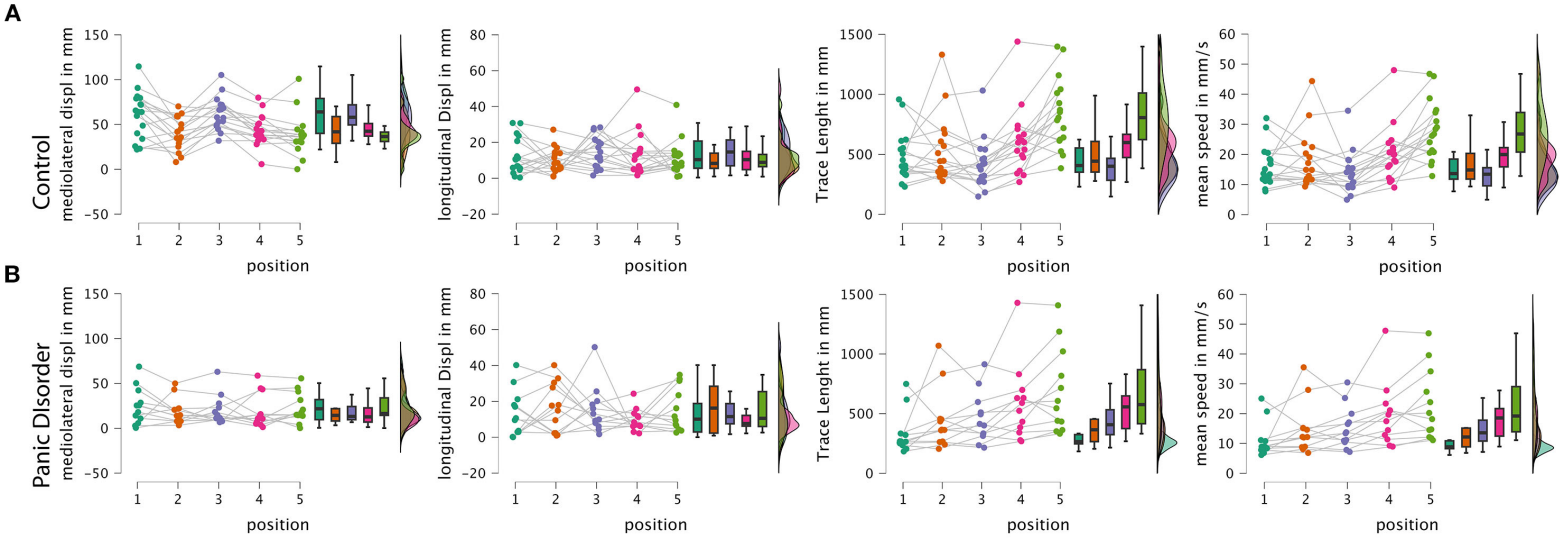
Raincloud plots from the RM-ANOVA. **(A)** Raincloud plots of the control group. **(B)** Raincloud plots of the panic disorder group. Displ, displacement; mm, millimeter; s, seconds; RM-ANOVA, repeated-measures ANOVA.

*Post-hoc* comparisons adjusted for multiple testing using both Bonferroni and Holm corrections revealed that the control group exhibited significantly higher mediolateral displacement across conditions, with an average difference of 28.13 mm (Cohen’s d = 1.45; pBonf < 0.001; pHolm < 0.001). When comparing positions independently, the greatest group differences occurred in conditions P1 (eyes open and firm surface) and P3 (eyes closed and firm surface), both showing highly significant differences (p < 0.001) favoring the control group, with large effect sizes (d > 1.88) (**Figure 3**).

Importantly, the simple main effects analysis showed that the control group exhibited a significant modulation of postural sway across conditions (F = 13.24; p < 0.001), while the PD group did not show significant differences across positions (F = 0.52; p = 0.719), suggesting that individuals with panic disorder employed a relatively rigid postural strategy regardless of the sensory demands. Specifically, only the control group showed significant reductions in sway from positions P1 and P3 to the more challenging foam-based conditions (P2, P4, and P5), consistent with a flexible adaptation to proprioceptive disruption.

### Anteroposterior CoP displacement

The same evaluation was conducted to analyze the mean anteroposterior CoP displacement. No significant main effects or interactions were observed for this variable. Specifically, there was no significant effect of condition (F = 0.64; p = 0.634; η^2^ = 0.014), group (F = 0.43; p = 0.518; η^2^ = 0.006), or condition-by-group interaction (F = 1.14; p = 0.344; η^2^ = 0.024). These results indicate that neither the type of sensory challenge nor group membership (control vs. panic disorder) significantly influenced anteroposterior sway. *Post-hoc* comparisons were not performed, given the absence of significant effects.

### Trace length

Analyzing the total path length (trace length) revealed a significant main effect of condition (F = 20.60; p < 0.001; η^2^ = 0.196), indicating that sensory manipulations affected overall postural sway. However, no significant main effect of group (F = 0.85; p = 0.364; η^2^ = 0.016) or group-by-condition interaction (F = 1.79; p = 0.135; η^2^ = 0.017) was found, suggesting that both groups exhibited similar overall trace length and similar patterns of change across conditions.

*Post-hoc* comparisons adjusted for multiple testing using both Bonferroni and Holm correction showed an interaction of condition and group. Condition P5 (eyes closed, foam surface without visual input, and cognitive load), for the control group, differed significantly from all other positions, with P5 showing the highest sway values. In the panic disorder group, positions P1, P2, and P3 differed significantly from P5, again reflecting increased sway under the most challenging sensory condition. Additionally, for intergroup comparisons comparing P4 (eyes closed and foam surface) to P5 (eyes closed, foam surface, and distraction), in the control group, the mean difference was −232.46 mm (Cohen’s d = −0.892; pBonf < 0.001; pHolm < 0.001); in the PD group, the mean difference was −98.52 mm (Cohen’s d = −0.378; pBonf 1.0; pHolm 0.309), highlighting the greater impact of distraction in the control group.

### Mean velocity

A similar pattern was observed for mean CoP velocity. Repeated-measures ANOVA revealed a significant main effect of condition (F = 20.84; p < 0.001; η^2^ = 0.197), with no significant group effect (F = 0.84; p = 0.368; η^2^ = 0.016) or group-by-condition interaction (F = 1.78; p = 0.138; η^2^ = 0.017). This indicates that mean velocity was primarily influenced by sensory condition, but not by diagnostic group, and that the pattern of response across conditions was similar in both groups.

*Post-hoc* tests confirmed that, in the control group, condition P5 resulted in significantly higher mean velocity compared to all other positions. Within the panic disorder group, mean velocity in P1, P2, and P3 was significantly lower than that in P5, showing that distraction had more impact on the control group.

*Post-hoc* analysis of both trace length and mean velocity revealed that in the control group, condition P5 differed from all other positions. In the panic disorder group, P1, P2, and P3 differed significantly from P5. For intergroup comparisons comparing P4 (eyes closed and foam surface) to P5 (eyes closed, foam surface, and distraction), in the control group, the mean difference was −7.765 mm/s (Cohen’s d = −0.894; pBonf < 0.001; pHolm < 0.001); in the PD group, the mean difference was −3.282 mm/s (Cohen’s d = −0.378; pBonf 1.0; pHolm 0.307), showing the same pattern as the trace length. The observed pattern mirrors that of the trace length.

### Machine learning classification analysis

In the evaluation of classification accuracy using machine learning models (regardless of testing position), Logistic Regression achieved the highest sensitivity (100%) and best AUC (96%), while the LDA model demonstrated the highest specificity (**Table 3**).

**Table 3:** Performance of machine learning models with default vs. Youden-optimized decision thresholds.

	Cutoff type	Cutoff value	Accuracy	Sensitivity	Specificity	PPV	NPV	F1-score	AUC
Decision Tree Classifier	Default	0.5	0.67	0.417	0.845	0.625	0.744	0.417	0.77
Decision Tree Classifier	Optimized	0.507	0.76	0.792	0.845	0.708	0.833	0.658	0.77
k-Nearest Neighbors Classifier	Default	0.5	0.667	0.75	0.662	0.492	0.775	0.557	0.787
k-Nearest Neighbors Classifier	Optimized	0.496	0.788	0.958	0.698	0.608	0.917	0.729	0.787
Linear Discriminant Analysis	Default	0.5	0.816	0.875	0.831	0.667	0.888	0.727	0.911
Linear Discriminant Analysis	Optimized	0.668	0.878	0.875	0.917	**0.875**	0.896	0.844	0.911
Logistic Regression	Default	0.5	0.785	0.875	0.795	0.625	0.888	0.686	**0.962**
Logistic Regression	Optimized	0.528	**0.938**	**1**	**0.908**	0.854	**1**	**0.914**	**0.962**
Random Forest Classifier	Default	0.5	0.819	0.833	0.881	0.75	0.858	0.733	0.914
Random Forest Classifier	Optimized	0.52	0.91	0.958	0.908	0.854	0.938	0.892	0.914

PPV, positive predictive value; NPV, negative predictive value; AUC, area under the ROC curve. Bold values represent the highest value for each collumm.

Receiver operating characteristic (ROC) curves for each cross-validation fold, as well as the mean ROC area, are available in the supplementary material.

Model-agnostic feature importance derived with LIME converged across classifiers, highlighting area-based sway metrics and mediolateral displacement as the most informative predictors (see **Table 4**). Specifically, equivalent area (mm^2^) was consistently ranked first for the linear models (LDA and Logistic Regression), whereas transversal (mediolateral) CoP displacement dominated the top positions for non-linear and instance-based methods (Random Forest, Decision Tree, and k-Nearest Neighbors).

**Table 4:** LIME ranking after training.

Ranking feature LIME	Models				
	Linear Discriminant Analysis	Logistic Regression	Random Forest Classifier	Decision Tree Classifier	k-Nearest Neighbors Classifier
1	Equivalent area (mmq)	Equivalent area (mmq)	Transversal CoP displacement (mm) [m.sd]	Transversal CoP displacement (mm) [m.sd]	Transversal CoP displacement (mm) [m.sd]
2	Transversal CoP displacement (mm) [m.sd]	Transversal CoP displacement (mm) [m.sd]	Longitudinal CoP displacement (mm) [m.sd]	LFS (1/cm)	Longitudinal CoP displacement (mm) [m.sd]
3	Fixed mean area (mmq)	Inertial axes (mm) [asseX.asseY]	Equivalent area (mmq)	Max radius (mm)	Peak number
4	Speed (mm/s) [m.sd]	Radius (mm) [m.sd]	Peak number	D spectrum. maximum peak and frequency (Hz)	Max radius (mm)
5	Variable mean area (mmq)	Peak number	Max radius (mm)	Min radius (mm)	LFS (1/cm)
6	Inertial axes (mm) [asseX.asseY]	Fixed mean area (mmq)	Fixed mean area (mmq)	Peak number	Equivalent area (mmq)
7	Peak number	Px spectrum. maximum peak and frequency (Hz)	D spectrum. maximum peak and frequency (Hz)	Longitudinal CoP displacement (mm) [m.sd]	Min radius (mm)
8	Radius (mm) [m.sd]	Max radius (mm)	Speed (mm/s) [m.sd]	Geographic area (mmq)	Regression angle:
9	Geographic area (mmq)	LFS (1/cm)	LFS (1/cm)	Px spectrum. maximum peak and frequency (Hz)	D spectrum. maximum peak and frequency (Hz)
10	Px spectrum. maximum peak and frequency (Hz)	Py spectrum. maximum peak and frequency (Hz)	Inertial axes (mm) [asseX.asseY]	Equivalent area (mmq)	Px spectrum. maximum peak and frequency (Hz)

LIME, Local Interpretable Model-Agnostic Explanations; CoP, center of pressure; mm, millimeters; mm^2^, square millimeters; Hz, hertz; Px/Py spectrum, maximum peak and peak frequency in the power spectrum of the CoP x/y component; D spectrum, maximum peak and peak frequency in the spectrum of the diagonal signal; LFS (1/cm), length-to-surface ratio; LDA, Linear Discriminant Analysis; K–NN, k–Nearest Neighbors.

Notably, the prominence of mediolateral displacement in the LIME rankings aligns with the RM–ANOVA finding of reduced mediolateral sway in PD, reinforcing the physiological plausibility of the classifiers.

## Discussion

These results support the central hypothesis of this study, revealing that individuals with panic disorder exhibit alterations in static postural control, showing the presence of distinct postural control strategies in individuals with PD, as evidenced by the differences in mediolateral CoP displacement. Furthermore, when evaluating the impact of a distracting cognitive activity, we observed a different pattern for the groups, marked by a greater effect in the control group compared to individuals with PD.

Extending this analysis with computational techniques based on artificial intelligence (AI), we observed a remarkable capacity to classify subjects accurately using the postural static pattern.

### Postural control differences between groups

One of the main findings was a reduction in mediolateral CoP displacement in the PD group compared to the control group, indicating a smaller amplitude of lateral sway. A marked difference was also observed in response to the use of foam surfaces, which reduce proprioceptive perception: while the control group demonstrated notable changes in conditions P2 and P4 (performed on foam), individuals with PD adopted a strategy characterized by postural rigidity and reduced mediolateral displacement in all the conditions. This behavior is likely a defensive mechanism aimed at preventing or minimizing the subjective sensation of imbalance or vertigo, consistent with the “surface dependence” model previously described in individuals with anxiety and PD (7).

In the variables trace length and CoP velocity, condition P5 (eyes closed, foam surface, and distraction) revealed the greatest distinction within groups, representing the most challenging scenario in both sensory and cognitive terms.

These results suggest that individuals with PD exhibit greater difficulty in postural adaptation when exposed to higher sensory and attentional demands. This behavior is consistent with existing literature on postural control alterations in challenging environments, where sensory cues are reduced or conflicting in individuals with PD (7, 16, 17, 21).

Condition P5, combining high postural demand with cognitive distraction, created a maximally challenging context through the concurrent removal of sensory cues and imposition of attentional load. This scenario was intended to reduce postural rigidity under pressure. In both intra- and intergroup comparisons, we observed an increased differentiation in trace length and speed, suggesting that distraction-based strategies may influence postural control mechanisms.

### Machine learning methods for physiological data analysis

ML models demonstrated robust performance, particularly for Logistic Regression under an optimized cutoff: sensitivity 100.0%, specificity 90.8%, accuracy 93.8%, F1 = 0.914, and AUC = 0.962. LDA retained the highest specificity (91.7%), and Random Forest also exhibited high accuracy (91.0%) with a sensitivity of 95.8%. These models enable integrated evaluation of the 27 variables extracted from the SWAY system and offer a clear advantage for assessing a complex phenomenon such as postural control, as they do not rely on assumptions of feature independence or linearity.

Among other potential biomarkers, hematological alterations such as platelet distribution width (PDW) have shown promising results, with a sensitivity of 85%, specificity of 79%, and an AUC of 89% (30).

In contrast, conventional panic disorder screening and assessment tools appear less effective than posturographic evaluation. The PHQ-PD, developed as a screening tool, demonstrates 77% sensitivity, 72% specificity, and an AUC of 79% ²^5^. The PDSS, a severity assessment tool, yields 83.3% sensitivity, 64% specificity, and an AUC of 79% (25).

To make the models more transparent and explainable, we used LIME, a simple post–hoc, model-agnostic tool that shows which stabilometric features contributed most to each prediction. LIME helps clinicians see a plausible rationale behind the output, but its explanations should be read with caution. They can vary across runs or small input changes, depend on user-chosen settings, and are an approximation of the original model rather than the model itself. Because of this, we i) fixed random seeds and reported LIME parameters, ii) checked whether explanations are consistent when we repeated the analysis, and iii) avoided causal claims using LIME only as supportive evidence. Overall, LIME helped communicate which posturographic features tended to influence the classifier, and we explicitly treated it as one piece of an explainable ML toolkit, not a definitive one (31–33).

These findings align with and extend previous literature applying machine learning techniques to physiological and behavioral markers in mental health. Previous work has highlighted how ML can reveal complex biomechanical patterns not captured by traditional statistics (22). In panic disorder, prior studies have reported altered balance strategies and surface dependence (7, 15), findings that our results validate by showing reduced mediolateral sway and a rigid postural strategy in PD patients. Furthermore, posturography has been shown to detect subtle abnormalities in psychiatric populations (17–19), but without leveraging ML for classification. By contrast, our work demonstrates that integrating ML models with posturographic data markedly improves discrimination, outperforming conventional clinical tools such as the PHQ-PD (29) and the PDSS (25). Compared with other proposed biomarkers, such as platelet indices in panic disorder (30), our models achieved higher accuracy and AUC values. Taken together, these results highlight the novelty and clinical utility of ML-enhanced posturography as an objective adjunct to established diagnostic scales.

## Clinical and public health implications

The identification of distinctive static postural control alterations in individuals with PD highlights the potential for objective physiological markers to complement traditional diagnostic and monitoring approaches in mental health. The use of stabilometric assessment, particularly when integrated with machine learning models, demonstrates high sensitivity and specificity in discriminating between PD patients and healthy controls, outperforming conventional screening tools. This suggests that objective posturographic data could serve as a valuable adjunct in the clinical evaluation of anxiety disorders, facilitating earlier detection.

From a public health perspective, the implementation of such low-cost, non-invasive physiological markers could enhance large-scale screening, monitoring, and preventive initiatives for anxiety disorders, ultimately reducing the burden of untreated or misdiagnosed cases and improving overall mental health outcomes. Nevertheless, further studies are warranted to externally validate these findings. Additionally, the development and validation of portable and user-friendly devices for the assessment of static postural control are necessary to enable the widespread clinical application of these biomarkers in routine mental health care.

## Limitations

The study’s limitations include the relatively small sample size, which led to the decision to apply ML learning reinforcement through repeated measures in the PD group and control group, increasing the risk of overfitting. Additionally, a potential selection bias may have been introduced due to the spontaneous recruitment of participants via online flyer distribution, which prevented the stratification and exclusion of individuals using pharmacological treatments or with psychiatric comorbidities such as generalized anxiety disorder (GAD) or major depressive disorder (MDD).

Taken together, these limitations qualify our findings as promising but preliminary. Addressing them via external validation, richer task batteries, stricter control of potential confounders, probability calibration, and complementary explainability will be essential to move toward a clinically reliable, generalizable tool for posturography-based assessment in panic disorder.

## Conclusion

Posturographic analysis in individuals with panic disorder revealed consistent findings, and the application of machine learning methods significantly enhanced the classification power of this physiological marker.

Future efforts should aim to expand the sample and validate these findings externally. Moreover, developing more practical and accessible methods for assessing postural control in clinical settings, without reliance on force platforms, remains a crucial objective.

## Data Availability

The datasets and code generated and analyzed during the current study are intellectual property of Infinity Doctor’s Inc. and cannot be made publicly available. However, anonymized datasets and the machine learning model code may be shared with qualified researchers upon reasonable request to the corresponding author, subject to company approval and compliance with ethical and privacy regulations.

## Supplementary material

Supplementary Table 1 Hyperparameter search space and optimal settings for supervised classifiers. The Hyperparameter Space column lists the ranges explored for each model; the Best Hyperparameters column reports the best-performing configuration selected by the inner CV procedure. Standardization was applied to the data for LDA, LR, and KNN before tuning. LDA, Linear Discriminant Analysis; LR, Logistic Regression; RF, Random Forest; DT, Decision Tree; KNN, k-Nearest Neighbors; CV, cross-validation; AUC, area under the ROC curve; C, inverse regularization strength in LR; p, Minkowski distance parameter (p=1 Manhattan, p=2 Euclidean).

Supplementary Figure 1 ROC curves — Decision Tree. Left: ROC curves for each fold (AUCs: 0.833, 0.667, 0.714, 0.867). Right: mean ROC across folds (AUC = 0.771 ± 0.082; n = 4). The diagonal dashed line indicates the random classifier (AUC = 0.5); ROC, receiver operating characteristic; AUC, area under the curve.

Supplementary Figure 2 ROC curves — K–Nearest Neighbors (KNN). Left: ROC curves for each fold (AUCs: 0.833, 0.722, 0.857, 0.733). Right: mean ROC (AUC = 0.787 ± 0.059; n = 4). The diagonal dashed line indicates the random classifier (AUC = 0.5). ROC, receiver operating characteristic; AUC, area under the curve.

Supplementary Figure 3 ROC curves — Linear Discriminant Analysis (LDA). Left: ROC curves for each fold (AUCs: 0.833, 0.944, 1.000, 0.867). Right: mean ROC (AUC = 0.912 ± 0.065; n = 4). The diagonal dashed line indicates the random classifier (AUC = 0.5). ROC, receiver operating characteristic; AUC, area under the curve;

Supplementary Figure 4 ROC curves — Logistic Regression. Left: ROC curves for each fold (AUCs: 0.917, 1.000, 1.000, 0.933). Right: mean ROC (AUC = 0.963 ± 0.038; n = 4). The diagonal dashed line indicates the random classifier (AUC = 0.5). ROC, receiver operating characteristic; AUC, area under the curve.

Supplementary Figure 5 ROC curves — Random Forest. Left: ROC curves for each fold (AUCs: 0.833, 0.889, 1.000, 0.933). Right: mean ROC (AUC = 0.914 ± 0.061; n = 4). The diagonal dashed line indicates the random classifier (AUC = 0.5). ROC, receiver operating characteristic; AUC, area under the curve.

## References

[1] Grassi M Caldirola D Di Chiaro NV Riva A Daccò S Pompili M. Are respiratory abnormalities specific for panic disorder? A meta-analysis. Neuropsychobiology. (2014) 70:52–60. doi: 10.1159/000364830, PMID: 25247676

[2] Takakusaki K. Functional neuroanatomy for posture and gait control. J movement Disord. (2017) 10:1–17. doi: 10.14802/jmd.16062, PMID: 28122432 PMC5288669

[3] Caldirola D Bellodi L Caumo A Migliarese G Perna G. Approximate entropy of respiratory patterns in panic disorder. Am J Psychiatry. (2004) 161:79–87. doi: 10.1176/appi.ajp.161.1.79, PMID: 14702254

[4] Meuret AE Rosenfield D Wilhelm FH Zhou E Conrad A Ritz T. Do unexpected panic attacks occur spontaneously? Biol Psychiatry. (2011) 70:985–91. doi: 10.1016/j.biopsych.2011.05.027, PMID: 21783179 PMC3327298

[5] Perna G Iannone G Torti T Caldirola D. Panic disorder, is it really a mental disorder? In: From body functions to the homeostatic brain, in Panic Disorder. Springer International Publishing, Cham (2016). p. 93–112. doi: 10.1007/978-3-319-12538-1_4

[6] Kim CH Nguyen SA. Anxiety and depression in adults with vestibular disorders: A systematic review and meta-analysis. Laryngoscope. (2025) 135:1–12. doi: 10.1002/lary.70055, PMID: 40815545 PMC12793953

[7] Jacob RG. Surface dependence: a balance control strategy in panic disorder with agoraphobia. Psychosomatic Med. (1997) 59:323–30. doi: 10.1097/00006842-199705000-00016, PMID: 9178344

[8] Bronstein A Brandt T. Clinical disorders of balance, posture and gait, 2Ed. London: CRC Press (2004). Available online at: https://books.google.com/books/about/Clinical_Disorders_of_Balance_Posture_an.html?hl=&id=OUFZDwAAQBAJ (Accessed September 29, 2025).

[9] McCray LR Kim CH Nguyen SA Harvey EA Meyer TA. Panic disorder in patients with vestibular dysfunction: A systematic review and meta-analysis. Otology Neurotology. (2025) 46:621–6. doi: 10.1097/MAO.0000000000004506, PMID: 40210233

[10] Balaban CD Thayer JF. Neurological bases for balance-anxiety links. J Anxiety Disord. (2001) 15:53–79. doi: 10.1016/s0887-6185(00)00042-6, PMID: 11388358

[11] Ivanenko Y Gurfinkel VS. Human postural control. Front Neurosci. (2018) 12:171. doi: 10.3389/fnins.2018.00171, PMID: 29615859 PMC5869197

[12] Takakusaki K. Forebrain control of locomotor behaviors. Brain Res Rev. (2008) 57:192–8. doi: 10.1016/j.brainresrev.2007.06.024, PMID: 17764749

[13] Kyriakoulis P Wijaya C Quagliato L Freire RC Nardi AE. Neurocircuitry and neuroanatomy in panic disorder: A systematic review. Alpha Psychiatry. (2025) 26:38756. doi: 10.31083/AP38756, PMID: 40110375 PMC11916057

[14] Duarte M Freitas SMSF. Revision of posturography based on force plate for balance evaluation. Revista brasileira de fisioterapia. (2010) 14(3):183–92. doi: 10.1590/S1413-35552010000300003, PMID: 20730361

[15] Jacob RG Redfern MS Furman JM. Space and motion discomfort and abnormal balance control in patients with anxiety disorders. J neurology neurosurgery Psychiatry. (2009) 80:74–8. doi: 10.1136/jnnp.2007.136432, PMID: 18653552 PMC4893779

[16] Boffino CC de Sá CS Gorenstein C Brown RG Basile LF Ramos RT. Fear of heights: cognitive performance and postural control. Eur Arch Psychiatry Clin Neurosci. (2009) 259:114–9. doi: 10.1007/s00406-008-0843-6, PMID: 18806914

[17] Caldirola D Teggi R Bondi S Lopes FL Grassi M Bussi M. Is there a hypersensitive visual alarm system in panic disorder? Psychiatry Res. (2011) 187:387–91. doi: 10.1016/j.psychres.2010.05.012, PMID: 21477868

[18] Forghieri M Monzani D Mackinnon A Ferrari S Gherpelli C Galeazzi GM. Posturographic destabilization in eating disorders in female patients exposed to body image related phobic stimuli. Neurosci Lett. (2016) 629:155–9. doi: 10.1016/j.neulet.2016.07.002, PMID: 27397012

[19] Pimentel BN Santos Filha VAVD. Occurrence of psychiatric conditions, use of psychotropic medications and its relationship with postural balance in subjects with dizziness. CoDAS. (2019) 31:e20180111. doi: 10.1590/2317-1782/20182018111, PMID: 31271579

[20] Perna G Alpini D Caldirola D Raponi G Cesarani A Bellodi L. Serotonergic modulation of the balance system in panic disorder: an open study. Depression Anxiety. (2003) 17:101–6. doi: 10.1002/da.10092, PMID: 12621600

[21] Yardley L Britton J Lear S Bird J Luxon LM. Relationship between balance system function and agoraphobic avoidance. Behav Res Ther. (1995) 33:435–9. doi: 10.1016/0005-7967(94)00060-w, PMID: 7755529

[22] Breiman L. Machine learning. Dordrecht: Springer (2001) 45:5–32. doi: 10.1023/a:1010933404324.

[23] Halilaj E Rajagopal A Fiterau M Hicks JL Hastie TJ Delp SL. Machine learning in human movement biomechanics: Best practices, common pitfalls, and new opportunities. J biomechanics. (2018) 81:1–11. doi: 10.1016/j.jbiomech.2018.09.009, PMID: 30279002 PMC6879187

[24] First MB Williams JBW Karg RS Spitzer RL. Entrevista Clínica Estruturada para os Transtornos do DSM-5: SCID-5-CV Versão Clínica. Porto Alegre: Artmed Editora (2017). Available online at: https://play.google.com/store/books/details?id=qPcoDwAAQBAJ.

[25] Shear MK Rucci P Williams J Frank E Grochocinski V Vander Bilt J. Reliability and validity of the Panic Disorder Severity Scale: replication and extension. J Psychiatr Res. (2001) 35:293–6. doi: 10.1016/s0022-3956(01)00028-0, PMID: 11591432

[26] BTS Bioengineering S.p.A. P-6000 digital force plate . Available online at: https://www.btsbioengineering.com/products/p-6000/ (Accessed 29 August 2025).

[27] BTS Bioengineering (BTS S.p.A.). SWAY, release 1.4.10.7 [computer software]. Amsterdam: JASP Team (2003).

[28] JASP Team. JASP (Version 0.95.1)[Computer software]. (2025).

[29] Muñoz-Navarro R Cano-Vindel A Wood CM Ruíz-Rodríguez P Medrano LA Limonero JT. The PHQ-PD as a screening tool for panic disorder in the primary care setting in Spain. PloS One. (2016) 11:e0161145. doi: 10.1371/journal.pone.0161145, PMID: 27525977 PMC4985125

[30] Ransing RS Gupta N Agrawal G Mahapatro N. Platelet and red blood cell indices in patients with panic disorder: A receiver operating characteristic analysis. J Neurosci Rural Pract. (2020) 11:261–6. doi: 10.1055/s-0040-1703422, PMID: 32367981 PMC7195955

[31] Ribeiro MT Singh S Guestrin C. Why should I trust you?: explaining the predictions of any classifier. In: Proceedings of the 22nd ACM SIGKDD international conference on knowledge discovery and data mining New York: Association for Computing Machinery (2016). p. 1135–44. doi: 10.1145/2939672.2939778

[32] Garreau D von Luxburg U. Explaining the explainer: A first theoretical analysis of LIME. In: Proceedings of the 23rd international conference on artificial intelligence and statistics (AISTATS), vol. 108. Brookline, MA: PMLR (2020). p. 1287–96.

[33] Burger C Chen L Le T. Are your explanations reliable? Investigating the stability of LIME in explaining text classifiers by marrying XAI and adversarial attack. In: Proceedings of the 2023 conference on empirical methods in natural language processing (EMNLP) Stroudsburg, PA: Association for Computational Linguistics (2023). p. 12831–44. doi: 10.18653/v1/2023.emnlp-main.792

