# Supplementary material for: Machine learning analysis of posturography in panic disorder: a pilot study for objective physiological biomarker identification
Source: Front Psychiatry. 2025 Oct 16;16:1663556. doi: 10.3389/fpsyt.2025.1663556 (PMC12573136; doi:10.3389/fpsyt.2025.1663556)
Supplement: Supplementary Table 1 — Hyperparameter search space and optimal settings for supervised classifiers. The Hyperparameter Space column lists the ranges explored for each model; the Best Hyperparameters column reports the best-performing configuration selected by the inner CV procedure. Standardization was applied to the data for LDA, LR, and KNN before tuning. LDA, Linear Discriminant Analysis; LR, Logistic Regression; RF, Random Forest; DT, Decision Tree; KNN, k-Nearest Neighbors; CV, cross-validation; AUC, area under the ROC curve; C, inverse regularization strength in LR; p, Minkowski distance parameter (p=1 Manhattan, p=2 Euclidean). [file Table1.docx]

| Models | Hyperparameter Space | Best Hyperparameters |
| --- | --- | --- |
| Linear Discriminant Analysis | **solver**: eigen, lsqr, svd  **tolerance**: (1e-5,1e-1)  **shrinkage**: (1e-5, 1e-4,1e-3,1e-2,1e-1,1,0) | **solver**: eigen  **tolerance**: 0.0004009279557990464  **shrinkage**:0.0001 |
| Logistic Regression | **solver**: liblinear, newton-cg, lbfgs  **penalty: L1, L2;**  **max iterations**: 100,1000  **C: 0.001,100** | **solver**: liblinear, newton-cg, lbfgs;  **penalty: L1, L2;**  **max iterations**: 100, 1000;  **C: 0.001,100** |
| Random Forest Classifier | **Number estimators**: 10, 100  **Maximum depth**:3, 30  **Minimum samples split**: 2, 10  **Minimum sample leaf**: 1, 10 | **Number estimators**: 100  **Maximum depth**:15  **Minimum samples split**: 5  **Minimum sample leaf**: 8 |
| Decision Tree Classifier | **Maximum depth**: 3, 30  **Minimum samples split**: 2, 10  **Minimum sample leaf**: 1, 10 | **Maximum depth**:16  **Minimum samples split**: 10  **Minimum sample leaf**: 4 |
| K-Neighbors Classifier | **Number neighbors**: 1, 10  **weights**: distance, uniform  **p: 1(manhattan), 2(Eucliedean)** | **Number neighbors**: 10  **weights**: distance  **p: 1(manhattan)** |

Table S1. Hyperparameter search space and optimal settings for supervised classifiers

Table S1. The Hyperparameter Space column lists the ranges explored for each model; the Best Hyperparameters column reports the best-performing configuration selected by the inner CV procedure. Standardization was applied to the data for LDA, LR, and KNN before tuning. Abbreviations: LDA, Linear Discriminant Analysis; LR, Logistic Regression; RF, Random Forest; DT, Decision Tree; KNN, k-Nearest Neighbors; CV, cross-validation; AUC, area under the ROC curve; C, inverse regularization strength in LR; p, Minkowski distance parameter (p=1 Manhattan, p=2 Euclidean).
