# Supplementary figures and images for: Machine learning analysis of posturography in panic disorder: a pilot study for objective physiological biomarker identification
Source: Front Psychiatry. 2025 Oct 16;16:1663556. doi: 10.3389/fpsyt.2025.1663556 (PMC12573136; doi:10.3389/fpsyt.2025.1663556)

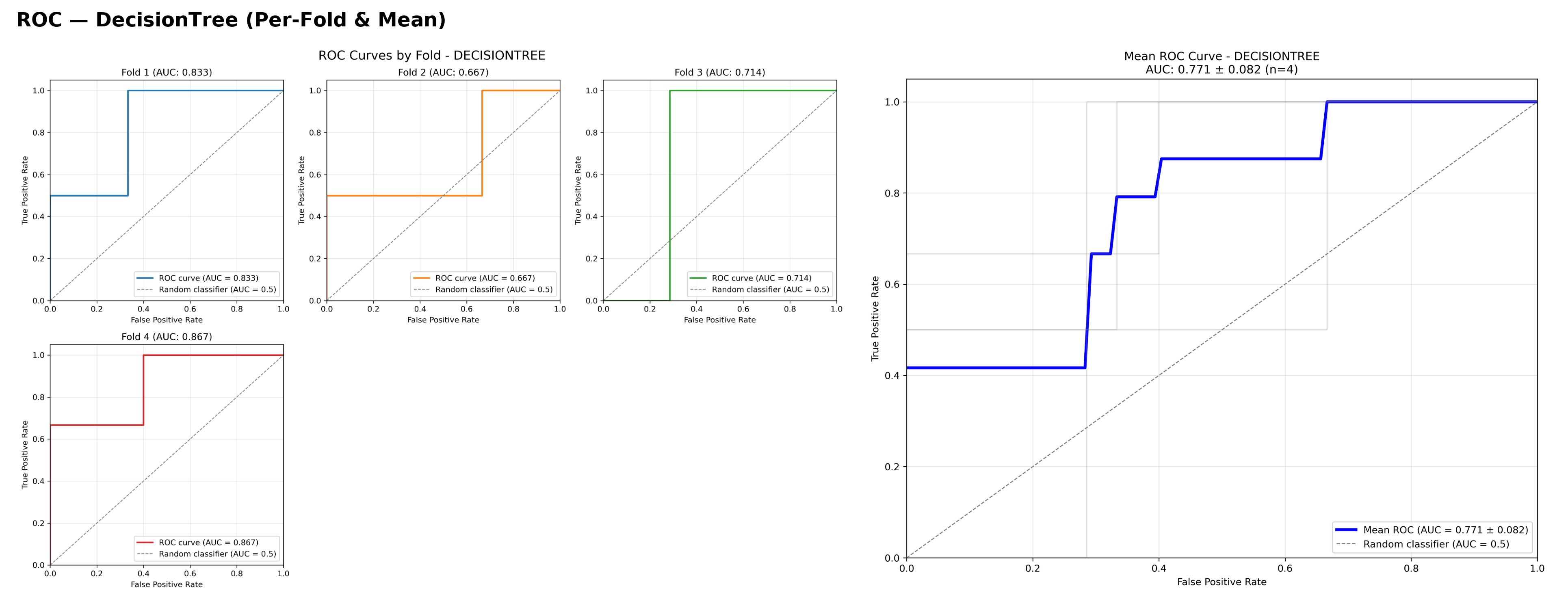

Supplement: Supplementary Figure 1 — ROC curves — Decision Tree. Left: ROC curves for each fold (AUCs: 0.833, 0.667, 0.714, 0.867). Right: mean ROC across folds (AUC = 0.771 ± 0.082; n = 4). The diagonal dashed line indicates the random classifier (AUC = 0.5); ROC, receiver operating characteristic; AUC, area under the curve. [file Image1.jpeg]

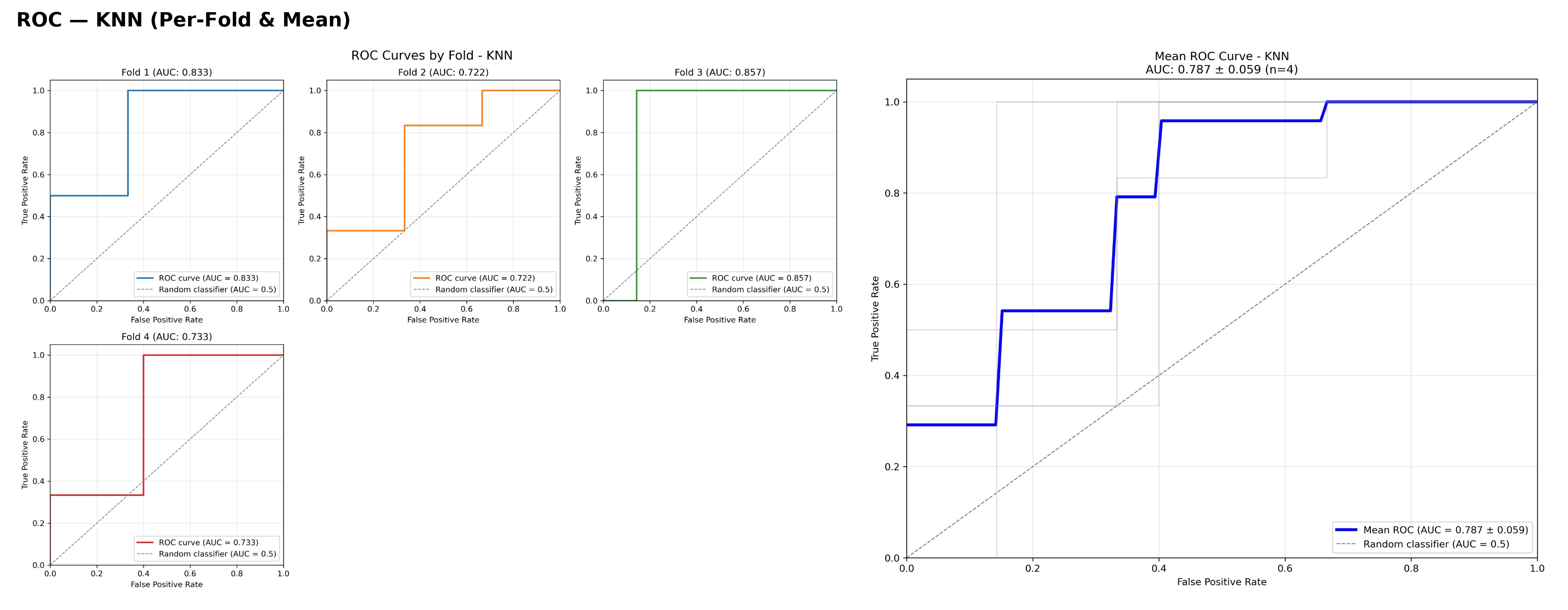

Supplement: Supplementary Figure 2 — ROC curves — K–Nearest Neighbors (KNN). Left: ROC curves for each fold (AUCs: 0.833, 0.722, 0.857, 0.733). Right: mean ROC (AUC = 0.787 ± 0.059; n = 4). The diagonal dashed line indicates the random classifier (AUC = 0.5). ROC, receiver operating characteristic; AUC, area under the curve. [file Image2.jpeg]

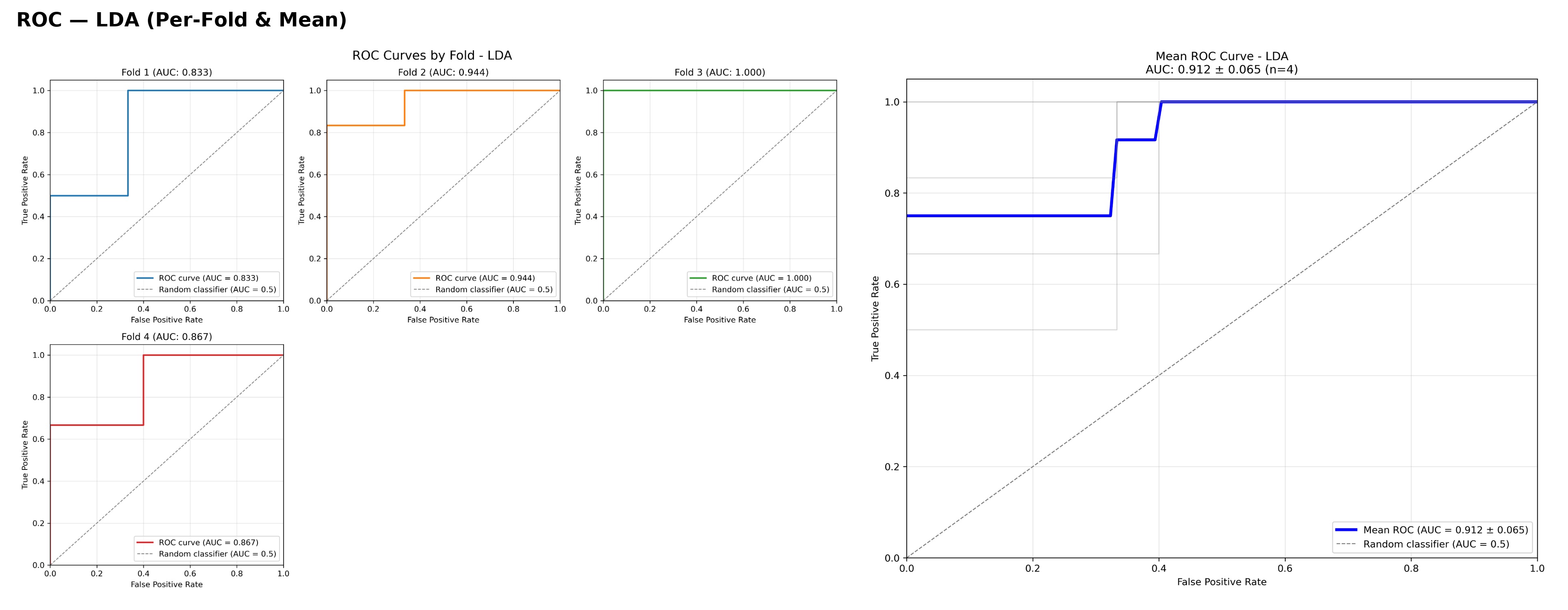

Supplement: Supplementary Figure 3 — ROC curves — Linear Discriminant Analysis (LDA). Left: ROC curves for each fold (AUCs: 0.833, 0.944, 1.000, 0.867). Right: mean ROC (AUC = 0.912 ± 0.065; n = 4). The diagonal dashed line indicates the random classifier (AUC = 0.5). ROC, receiver operating characteristic; AUC, area under the curve; [file Image3.jpeg]

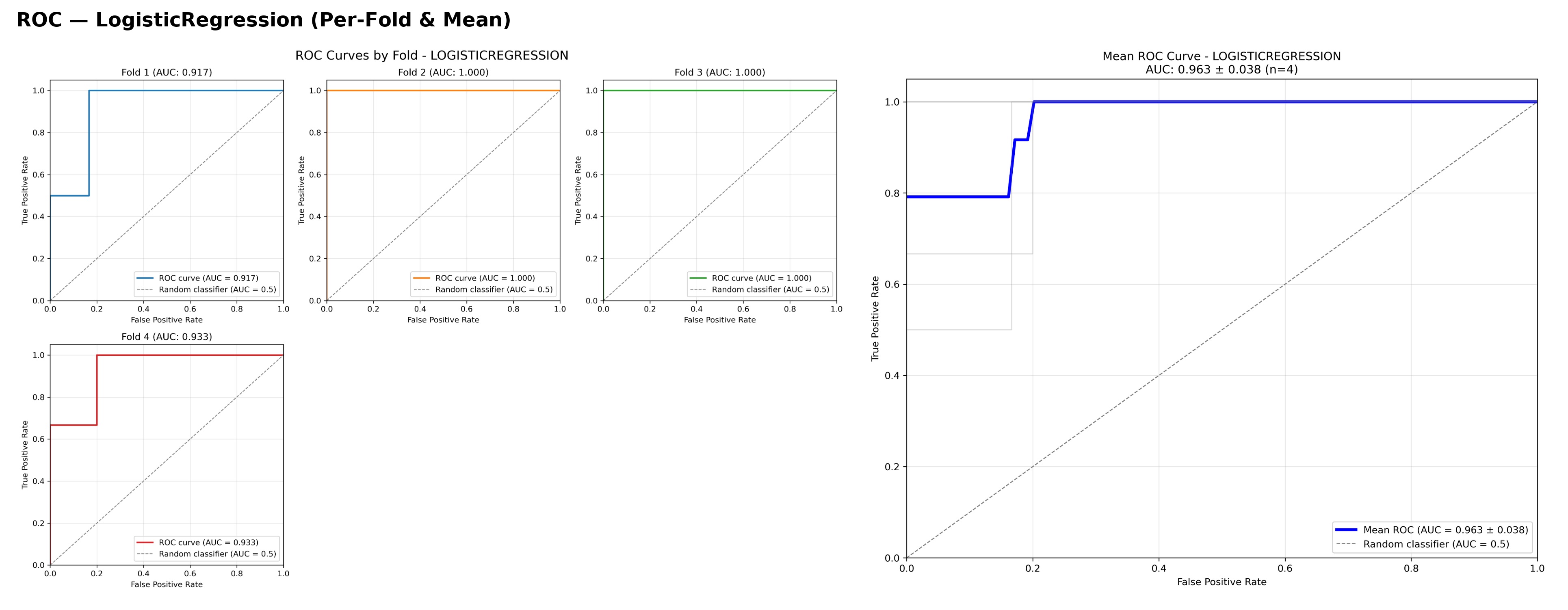

Supplement: Supplementary Figure 4 — ROC curves — Logistic Regression. Left: ROC curves for each fold (AUCs: 0.917, 1.000, 1.000, 0.933). Right: mean ROC (AUC = 0.963 ± 0.038; n = 4). The diagonal dashed line indicates the random classifier (AUC = 0.5). ROC, receiver operating characteristic; AUC, area under the curve. [file Image4.jpeg]

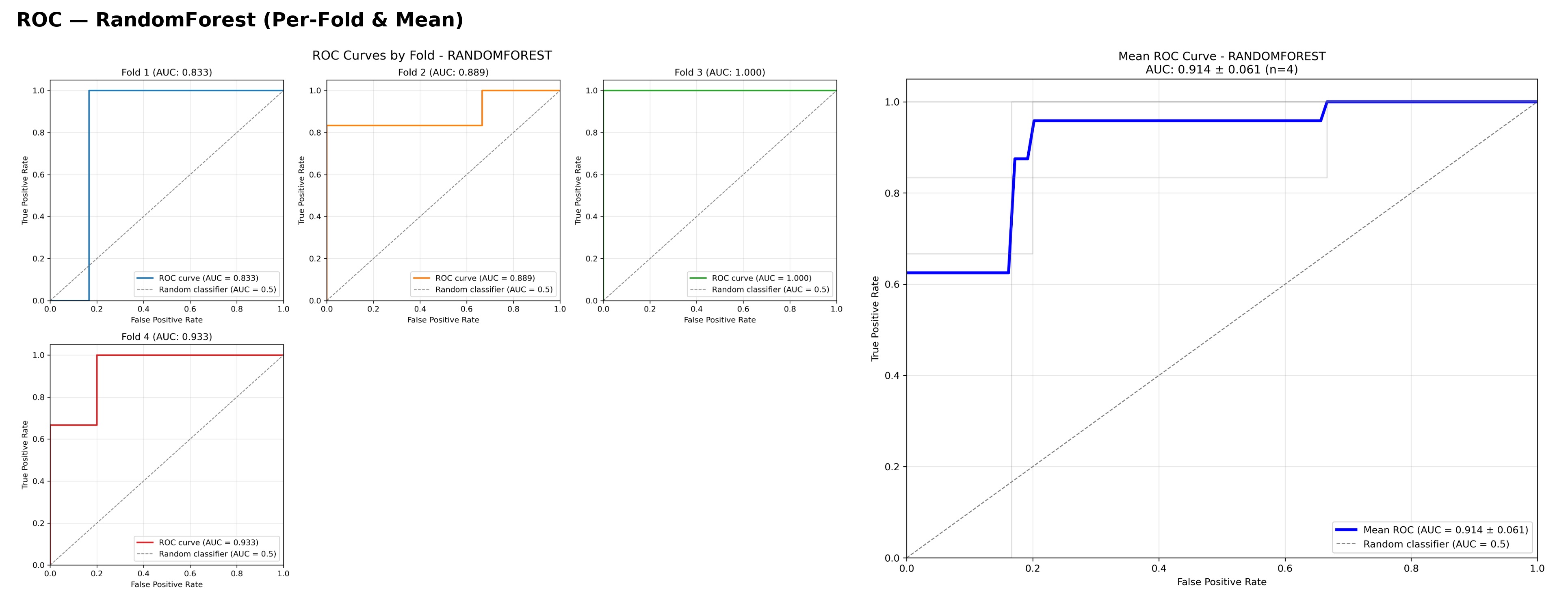

Supplement: Supplementary Figure 5 — ROC curves — Random Forest. Left: ROC curves for each fold (AUCs: 0.833, 0.889, 1.000, 0.933). Right: mean ROC (AUC = 0.914 ± 0.061; n = 4). The diagonal dashed line indicates the random classifier (AUC = 0.5). ROC, receiver operating characteristic; AUC, area under the curve. [file Image5.jpeg]
